# Health literacy competency requirements for health professionals: a Delphi consensus study in Taiwan

**DOI:** 10.1186/s12909-024-05198-4

**Published:** 2024-03-01

**Authors:** Han-Yi Tsai, Shoou-Yih D. Lee, Cliff Coleman, Kristine Sørensen, Tzu-I Tsai

**Affiliations:** 1 Department of Nursing, Taipei Veterans General Hospital, No.201, Sec. 2, Shipai Rd., Beitou District, Taipei City, 11217 Taiwan, ROC; 2 Department of Health Administration, College of Health Professions, Virginia Commonwealth University, 900 E. Leigh St. 8Th Floor, Box 980203, Richmond, VA 23298 USA; 3 Department of Family Medicine, Oregon Health & Science University, 3181 SW Sam Jackson Park Road, Portland, OR 97239 USA; 4 Global Health Literacy Academy, Borresøvej 26, 8240 Risskov, Denmark; 5 College of Nursing, National Yang Ming Chiao Tung University, Yang Ming Campus, No. 155, Sec. 2, Linong St. Beitou Dist., Taipei, 112304 Taiwan

**Keywords:** Consensus study, Modified Delphi, Health literacy competency, Health professionals

## Abstract

**Background:**

Cumulative evidence supports the importance of health literacy in determining the quality of healthcare delivery and outcomes. To enhance health literacy competencies among professionals and alleviate healthcare barriers owing to patients’ inadequate health literacy, evidence-based health literacy competency guidelines are needed for the development of health professionals’ training curricula. The aim of this study was to validate and refine a set of health literacy competencies, including knowledge, attitude, and skills of health professionals, and to prioritize the importance of health literacy practices among healthcare professionals.

**Methods:**

We employed a consensus-building approach that utilized a modified three-round Delphi process conducted in 2017. An online Delphi panel was assembled, comprising 20 Taiwanese health literacy experts from diverse fields such as medicine, nursing, public health, language, and communication. A set of health literacy competencies previously identified and validated by an international panel of health literacy experts was cross-culturally translated.

**Results:**

After three rounds of ratings and modifications, a consensus agreement was reached on 42 of 62 health literacy competencies, including 12 of 24 knowledge items, 9 of 11 attitude items, and 21 of 27 skill items. Of the 32 health literacy practices, “avoidance using medical jargon,” “speaking slowly and clearly with patients,” and “using analogies and examples” were deemed most important by the panelists.

**Conclusions:**

The Delphi panel’s consensus helped to identify a set of core health literacy competencies that could serve as measurable learning objectives to guide the development of a health literacy curriculum for health professionals. The prioritized health literacy practices can be employed as indicators of health literacy competencies that health professionals should learn and routinely use in clinical settings.

**Supplementary Information:**

The online version contains supplementary material available at 10.1186/s12909-024-05198-4.

## Introduction

Health literacy is related to individuals’ knowledge, motivation, and competencies to access, understand, appraise, and apply health information, and take appropriate decisions that are relevant to health promotion, disease prevention, and self-care management [1]. Because of research and advocacy by health literacy experts, inappropriate use of health services, poor health care quality, and adverse health are no longer considered as results of patients’ limited health literacy. Rather, healthcare providers and organizations are being held responsible for creating a “health literate” system that enables patients to find, understand, and use information to inform health-related decisions for themselves and their families.

Evidence suggests that healthcare providers equipped with health literacy and communication skills contribute positively to reducing health literacy related barriers and improving healthcare quality and patients’ health outcomes [2, 3]. However, studies have shown that health providers have a tendency to overestimate patients’ health literacy and they lack adequate health literacy competency to appropriately respond to and handle patients’ low health literacy issues [4, 5]. As medical technologies continue to advance and the healthcare delivery systems become more complex, increasing healthcare providers’ health literacy competency has become more crucial than ever [6, 7].

The Institute of Medicine has recommended health-related professional schools and professional continuing education programs, including medicine, dentistry, nursing, and other professionals, to develop health literacy curricula and training programs [8]. Similarly, many medical educators and researchers have urged the need to integrate health literacy training into the medical education curriculum [9]. These recommendations have thus far led to limited progress. Few undergraduate or continuing medical education programs have successfully incorporate health literacy in their curricula [6, 10]. Few curricular standards exist that address the need for health literacy training. With few exceptions where students may be exposed to health literacy concepts and practices in independent courses or during clinical rotations [2, 3], current learning models lack in-depth health literacy contents that included essential competencies and applications of health literacy principles [2, 5].

Coleman points out that a crucial challenge in integrating health literacy into existing health professional curricula is the lack of clear and widely accepted guidelines for defining and evaluating the contents of health literacy curricula across health professional programs [11]. Another challenge is that health-related professional schools and continuing education programs have limited resources, including instructional hours, financial resources, and faculty availability, to integrate health literacy into the full curriculum even when they acknowledge the importance of health literacy training [12]. As such, an evidence-based health literacy competency guideline that enlists and prioritizes a set of measurable core health literacy knowledge, attitudes, skills, and practices may enable health professional schools and programs to effectively design health literacy training programs and set clinical practice standards.

To rationally prioritize educational competencies for health literacy training, Coleman and colleagues [13] conducted an extensive literature review of health literacy competencies and practices and employed a Delphi consensus process to develop a set of measurable knowledge, skill, attitude, and practice elements to assess health professionals’ health literacy competencies. This set of educational competencies for health professionals has been refined and validated in the US and Europe [14, 15], and has led to prioritization of important health literacy practices [16]. Thus far, no similar work has been conducted in Asia.

In Taiwan, researchers surveyed nurses regarding their health literacy knowledge and the results showed that nurses answered merely half of the questions correctly [17]. Chang et al., (2016) and Chang et al. (2017) developed a health literacy assessment tool for health professionals. Their instrument was constructed predominately by combining items from several domestic surveys [18, 19]. Although the instrument may reflect specific contexts of the Taiwanese healthcare delivery system, it is focused on health literacy knowledge and does not take into account work by international scholars who have systematically identified health literacy competencies essential for health professionals. Moreover, the instrument was not designed for guiding the development of health literacy curricula.

The purpose of this study was to develop health literacy competencies for health professionals in Taiwan based on the work of Coleman and colleagues [13] and to prioritize the importance of health literacy practices in clinical settings. Our study results contribute to promoting cross-cultural application of health literacy competencies and facilitating the development of health literacy curricula in health professional education and training programs.

## Methods

We first translated the set of health literacy competencies developed by Coleman et al. [13], and then underwent a modified Delphi process.

### Translation of health literacy competencies for health professionals

We followed a standardized forward translation and back translation method developed by the World Health Organization to translate health literacy competencies from English to Chinese Mandarin [20]. Two native Chinese speakers, who were proficient in English, were involved in the forward translation; one had medical background and the other was a linguist. Each of them independently translated the set of health literacy competencies for health professionals [13]. They then discussed with the research team and compared their translations to reach a consensus on a final version of the translation.

Two native English speakers proficient in Chinese enlisted in the back translation. Similarly, one had medical and the other linguistic backgrounds. Without knowledge of the original wording of those health literacy competencies, each of them independently translated the Chinese version into English. Our research team then engaged all four translators to compare the two back-translated versions. Adjustments were made and a final version was agreed upon by everyone involved.

### A modified Delphi process

A three-round modified Delphi survey was applied in this study. The modified Delphi process was conducted anonymously and online.

#### Expert selection

Delphi experts were identified based on their expertise and leadership in health profession education in their respective fields as well as their knowledge of health literacy. Considering the short span of health literacy research in Taiwan (approximately 15 years), we employed the following eligibility criteria in the selection of Delphi experts: (1) the expert had three or more years of work experience in the field of health literacy and published at least one original article that listed them as a first author; (2) the expert had five or more years of work experience in the field of health literacy and had completed at least one systematic review article that listed them as a first author or corresponding author; or (3) the expert had been devoted to health literacy-related work for five or more years. De Villiers et al. (2005) suggested that the size of the expert panel for a Delphi study ranged from 15 to 30 [21]. In this study, 22 health literacy experts from various professional areas were invited, and 20 were finally recruited with purposive sampling to participate in 3 rounds of a Delphi process, The Delphi process was conducted in 2017.

#### Round 1 of Delphi

In Round 1 of the Delphi process, the expert panel was provided with 62 items of health literacy competencies to rate. Moreover, the expert panel was provided with the original and translated versions of items to review to ensure conceptual and cultural equivalence and to suggest changes [20, 22, 23].

The rating was focused on the appropriateness and importance of those items using a 4-point Likert-type scale ranging from 1 (very appropriate/important) to 4 (not appropriate/important). A *priori* cutoff point was set similar to the previous consensus level determined in the US and European studies, with consensus being defined as 70% or more of the expert panel agreement on the appropriateness and importance of an item [13, 14]. Items with less than 30% of agreement were removed. Experts were encouraged to recommend new items.

#### Round 2 of Delphi

During the second round, the experts received the quantitative results of the previous round – i.e., percentages of the panelists considering items as appropriate/important – along with anonymous suggestions. The expert panel was then asked to assess whether each item should be considered a core health literacy competency for health professionals. Items that at least 70% of the panelists considered core were retained. The panelists also discussed conceptual equivalence of the translation and the wording of each item. Items reflecting similar concepts were merged and necessary wording changes were made in accordance to the panelists’ suggestions.

During this round, the Q method was applied to prioritize the importance of health literacy practices based on their likely impact on patients. The aim of the analysis was to help healthcare providers and organizations ration their resources and time by focusing on health literacy practices that have the greatest impact. The Q method is a validated technique that has been applied to prioritize educational competencies for medical education [24]. In this study, the Q method followed the method used by Coleman et al. [13]. Each expert panel provided 32 items of health literacy practice and then placed each item in order of importance on the developed e-format Q sorting board.

#### Round 3 of Delphi

In the final round of consensus, the expert panel received the results of the retained competency items, the importance order of health literacy practices, and anonymous suggestions in the preceding rounds. The panelists scrutinized the final version and reached a consensus on health literacy competencies and practices. Although not a focus of this paper, the panelists also recommended additional work needed to turn health literacy competencies and practices into a measurable scale for evaluation purposes and suggested that the measurement could use a Likert-scale format.

### Data analysis

**Fig. 1:**
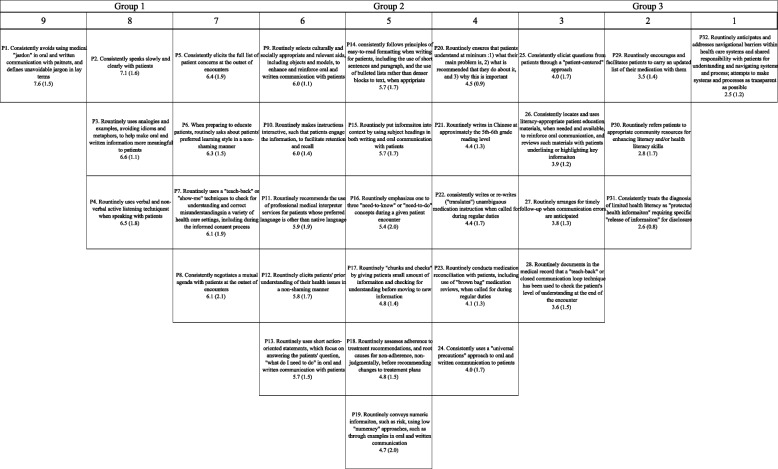
The ranking in the importance of health literacy practice. Footnote: The numbers represent mean ratings out of 9 and standard deviation in parentheses. The highest mean ratings of practice items on the left and the lowest mean ratings on the right

SPSS Version 22.0 and Microsoft Excel 2019 were utilized to analyze the quantitative data. Descriptive statistics, including means, standard deviations, and percentages, were applied. In implementing the Q method, we calculated the weighted mean scores of health literacy practice items and arranged them into a grid (Fig. 1), from highly important to least important.

### Ethical approval

The Institutional Board of the National Yang Ming University reviewed the protocol and approved the study through an expedited review (YM104142E).

## Results

A total of 20 experts participated in the Delphi study. Most of the experts were female (65%) and professors and researchers (65%). Their educational backgrounds included clinical medicine (25%), nursing (20%), pharmacy (10%), and public health (25%), and communication or linguistics (20%) (Table 1).

**Table 1:** Demographics characteristics of Delphi expert panel

Characteristics	N (%)
Gender	
Male	7 (35)
Female	13 (65)
Age	
40–49	9 (45)
50–59	6 (30)
60 or older	5 (25)
Professional Role	
Professor & Researcher	13 (65)
Clinical Practitioner	4 (20)
Manager	3 (15)
Education	
Health Professionals	16 (80)
Medicine	5 (25)
Nursing	4 (20)
Pharmacy	2 (10)
Public Health	5 (25)
Communication/Linguistic	4 (20)

Health literacy competencies retained after the Delphi process, including 12 knowledge items, 9 attitude items, and 21 skill items, are shown in Tables 2, 3, and 4, respectively. During round 1, 57 out of 62 (91.9%) health literacy competency items were accepted. The mean of the appropriateness rating by the panelists ranged from 1.15 to 2.70 and the mean of the importance rating ranged from 1.00 to 2.25 (Appendix Table 1). Based on the group consensus criterion, 22 out of 24 knowledge items, 10 out of 11 attitude items, and 25 out of 27 skill items were considered appropriate and important. No additional health literacy knowledge, attitudes, or skills were suggested in this round. In Round 2, 44 out of 57 items considered as core competencies achieved consensus (12 knowledge items, 9 attitude items, and 23 skill items). In Round 3, based on the panel’s feedback, 3 skill items that were similar were merged into 1. Overall, 42 competency items were retained, including 12 knowledge items, 9 attitude items, and 21 skill items.

**Table 2:** List of health literacy knowledge

Health literacy Knowledge
K1. knows that years of educational attainment is an inadequate marker for health literacy skills
K2. knows which kinds of words, phrases, or concepts may be jargon to patients
K3. knows that cultural and linguistic differences between patients and health care professionals can magnify health literacy issues
K4. knows that adults with low literacy tend to experience shame, and hide their lack of skills from health care professionals
K5. knows that “you can’t tell who has low health literacy by looking”
K6. recognizes “red flag” behaviors which may suggest a patient has low health literacy
K7. knows that health literacy is context-specific; individuals with high general literacy may have low health literacy
K8. knows that transition points, or “hand-offs” in health care (e.g., moving from in-patient to out-patient settings) are especially vulnerable to patient communication errors
K9. knows rationale for, and principles underpinning the need for a *universal precautions* approach to all health communication interactions
K10. knows best practice principles of plain language and clear health communication for oral and written communication
K11. knows examples of the direct relationship between health literacy and • knowledge about one’s chronic disease(s) and medications • adherence to medications and treatment plans • receipt of preventive health services • health outcomes or risk of harm
K12. recognizes potential legal implications for inadequately conveying health information to patients with low literacy or health literacy
Items not retained
• knows one or more definitions of health literacy • knows the basic literacy skill domains (reading, writing, speaking, listening, numeracy), and gives examples of health care related demands put on patients for each domain, including difficulties navigating health care systems • knows the difference between the ability to read, and reading comprehension, and why general reading levels do no not ensure patient understanding • estimates the prevalence of low literacy (or low health literacy) among adults, and knows that certain subgroups are at increased risk • knows that the average adult reads at an 8th–9th-grade reading level, but that most patient education materials are written at a much higher reading level • knows that tools are available for estimating individuals’ health literacy skills, but that routine screening for low health literacy has not been proven safe or acceptable • knows that health literacy may decrease during times of physical or emotional stress • knows that everyone, regardless of literacy level, benefits from and prefers clear plain language communication • knows that patients learn best when a limited number of new concepts are presented at any given time • recognizes potential legal implications for inadequately conveying health information to patients with low literacy or health literacy • knows that low health literacy has been associated with excess healthcare costs • knows that community resources exist for helping adults improve their general literacy skills

**Table 3:** List of health literacy attitude

Health Literacy Attitude
A1. expresses the attitude that effective communication is essential to the delivery of safe high-quality health care
A2. expresses the attitude that because the “culture” of healthcare includes special knowledge, language, logic, experiences and explanatory models of health and illness, every patient encounter can be considered a cross-cultural experience
A3. expresses acceptance of an ethical responsibility to facilitate the two-way exchange of information in “shared decision making” to the degree and at the level desired by the patient and their family
A4. acknowledges patients’ autonomous right to both informed consent, and “informed refusal” of recommended evaluations or treatments
A5. expresses empathy with patients’ potential sense of shame around low literacy (or health literacy) issues
A6. expresses a non-judgmental non-shaming respectful attitude toward individuals with limited literacy (or health literacy) skills
A7. expresses empathy with the common experience of the health care system as a confusing, stressful, frustrating, intimidating, and frightening physical and virtual environment for many patients
A8. expresses the attitude that every patient has the right to understand their health care, and that it is the health care professional’s duty to elicit and ensure patients’ best possible understanding of their health care
A9. expresses the attitude that it is a responsibility of all members of the healthcare team to be trained and proactive in addressing the communication needs of patients
Items not retained • exhibits the attitude that all patients are at risk for communication errors, and that one cannot tell who is at risk of communication errors simply by looking, or through typical health care interactions—a universal precautions approach is required with all patients • expresses the attitude that it is a responsibility of the health care sector to address the mismatch between patients’ and health care providers’ communication skills and tactics

**Table 4:** List of health literacy skill

Health Literacy Skill
S1. demonstrates ability to use common familiar lay terms, phrases and concepts, and appropriately define unavoidable jargon, and avoid using acronyms in oral and written communication with patients
S2. demonstrates ability to follow best-practice principles of easy-to-read formatting and writing in written communication with patients
S3. demonstrates the ability to put information into context by using subject headings in both written and oral communication with patients
S4. demonstrates ability to interpret or write information from a non-plain language format into a scientifically accurate 5th-6th grade reading level
S5. demonstrates ability to speak slowly and clearly with patients
S6. demonstrates ability to use verbal and non-verbal active listening techniques when speaking with patients
S7. demonstrates the ability to use action oriented statements to help patients know what they need to do
S8. demonstrates ability to select culturally and socially appropriate and relevant visual aids, including objects and models, to enhance and reinforce oral and written communication with patients
S9. demonstrates ability to make instructions interactive, such that patients engage the information, to facilitate retention and recall
S10. demonstrates ability to negotiate a mutual agenda for the encounter at the outset of the encounter
S11. demonstrates ability to elicit patients’ prior understanding of their health issues in a non-shaming manner (e.g., asks “what do you already know about high blood pressure?”)
S12. demonstrates ability to non-judgmentally elicit root causes of non-adherent health behaviors
S13. demonstrates effective use of a teach back or “show me” technique for assessing patients’ understanding
S14. demonstrates ability to effectively elicit questions from patients through a “patient-centered” approach (e.g., asks “what questions do you have?” rather than “do you have any questions?”)
S15. demonstrates ability to orally communicate accurately and effectively in patients’ preferred language, using medical interpreter services
S16. demonstrates ability to use written communication to reinforce important oral information
S17. demonstrates ability to emphasize one to three “need-to-know” or “need-to- do” concepts during a given patient encounter
S18. demonstrates the ability to convey numeric information, such as risk, using low numeracy approaches, such as through examples, in oral and written communication
S19. demonstrates ability to write or re-write (“translate”) unambiguous medication instructions (e.g., “take 1 tablet by mouth every morning and evening for high blood pressure,” rather than “take one tablet by mouth twice daily.”
S20. demonstrates ability to ask patients about their learning style preferences (e.g., ask patients, “what is the best way for you to learn new information?”
S21. demonstrates ability to use examples or analogies to improve patients’ comprehension
Items not retained • demonstrates ability to recognize, avoid and/or constructively correct the use of medical jargon, as used by others in oral and written communication with patients. (merged with S1) • demonstrates ability to recognize plain language principles in written materials produced by others. (merged with S4) • demonstrates ability to write in Chinese Mandarin at approximately the 5th-6th grade reading level. (merged with S4) • demonstrates ability to elicit the patient’s full set of concerns at the outset of the encounter. (merged with S14) • demonstrates ability to “Chunk and check” by giving patients small amounts of information and checking for understanding before moving to new information • demonstrates the ability to assess the usability of web-based patient resources

Figure 1 displays the result of the final version of Q sorts with the mean and standard deviation scores ranging from 7.6 (SD ± 1.5) to 2.5 (SD ± 1.2). The Q-sort grid showed the highest mean ratings of practice items on the left and the lowest mean ratings on the right. Additionally, we calculated the number and percentage of 20 experts who rated each of the items in Group 1 at an important level of ≥ 7 to check whether mean item ratings could be the result of an outlier. The findings showed the percentage ranging between 45 and 70%, suggesting little influence from outlier opinions. Table 5 shows the original and translated versions of health literacy practices in order of importance. “Avoiding using medical jargon” was ranked as the most important health literacy practice, followed by “speaking slowly and clearly with patients” and “using analogies and examples, avoiding idioms and metaphors, to help make oral and written information more meaningful to patients.”

**Table 5:** Health literacy practice ranking

Rank	Health Literacy Practice	Translate to Chinese, Mandarin (Traditional)
1	Consistently avoids using medical “jargon” in oral and written communication with patients. And defines unavoidable jargon in lay terms	與病患進行口頭或書面溝通時, 避免使用醫學術語; 對無法避免的專業術語, 會用淺顯易懂的話語解釋給病患聽
2	Consistently speaks slowly and clearly with patients	一直保持緩慢而清楚的方式與病患說話
3	Routinely uses analogies and examples, avoiding idioms and metaphors, to help make oral and written information more meaningful to patients	提供書面和口頭資訊時, 為了讓病患更清楚瞭解, 能常規地使用比喻或舉例, 避免使用成語或隱喻
4	Routinely uses verbal and non-verbal active listening techniques when speaking with patients	在與病患溝通時, 能常規地使用積極性語言及非語言的聆聽技巧
5	Consistently elicits the full list of patient concerns at the outset of encounters	從初次與病患會面互動, 能持續引導病患列出其所有的擔憂
6	When preparing to educate patients, routinely asks about patients’ preferred learning style in a non-shaming manner (e.g., asks “what is the best way for you to learn new information?”)	對病患做健康指導時, 能常規地使用不讓病患感到羞愧的問法來了解其偏好的學習模式 (例如: 你都用甚麼方式學習新東西?)
7	Routinely uses a “teach-back” or “show-me” techniques to check for understanding and correct misunderstandings in a variety of health care settings, including during the informed consent process	在諸多健康照護情境下, 包括獲得知情同意的過程中, 持續地應用「回覆示教」(teach-back) 或「示範給我看」(show-me) 的技巧, 來確認病患的瞭解程度並糾正誤解
8	Consistently negotiates a mutual agenda with patients at the outset of encounters	從初次與病患會面互動, 能持續與病患討論出醫病共同決定的治療計畫
9	Routinely selects culturally and socially appropriate and relevant aids, including objects and models, to enhance and reinforce oral and written communication with patients	常規地選用符合社會風俗民情的輔助工具, 包括物品或模型, 來加強與病患的口頭或書面溝通
10	Routinely makes instructions interactive, such that patients engage the information, to facilitate retention and recall	常規地給予病患互動性的指導, 有助於病患了解訊息, 進而促進對訊息的記憶與回溯
11	Routinely recommends the use of professional medical interpreter services for patients whose preferred language is other than English	對於那些不善用本國語言的病患, 能常規地建議其使用專業醫療翻譯服務
12	Routinely elicits patients’ prior understanding of their health issues in a non-shaming manner (e.g., asks “what do you already know about high blood pressure?”)	能常規地使用不讓病患感到羞愧的問法來引導病患描述對自己健康狀況的了解 (例如: 你對高血壓的了解是什麼?)
13	Routinely uses short action-oriented statements, which focus on answering the patients’ question, “what do I need to do” in oral and written communication with patients	與病患用書面和口頭方式溝通時, 能常規地採用「行動為導向」的簡要陳述來回答病患所提出「我需要做甚麼」的疑問
14	Consistently follows principles of easy-to-read formatting when writing for patients, including the use of short sentences and paragraph, and the use of bulleted lists rather than denser blocks to text, when appropriate	遵循易於閱讀格式的原則來書寫給病患的資訊, 包括使用簡短的語句和段落, 以及條列式重點取代長篇幅的文字陳述
15	Routinely puts information into context by using subject headings in both written and oral communication with patients	與病患用書面和口頭方式溝通時, 能常規地使用標題, 讓病患更容易了解主題內容
16	Routinely emphasizes one to three “need-to-know” or “need-to-do” concepts during a given patient encounter	在每次與病患的會面互動, 能常規性的強調一到三個「需要知道」或「需要做」的概念
17	Routinely “chunks and checks” by giving patients small amounts of information and checking for understanding before moving to new information	持續地使用「分段確認」 (chunks and checks) 的技巧, 把要跟病患溝通的訊息分成幾個部分說明, 確認病患瞭解部分後, 再給予新的訊息
18	Routinely assesses adherence to treatment recommendations, and root causes for non-adherence, non-judgmentally, before recommending changes to treatment plans	在建議更改治療計畫前, 會常規地評估病患對原治療建議的依從性, 並以客觀的角度了解造成病患不依從的根本原因
19	Routinely conveys numeric information, such as risk, using low “numeracy” approaches, such as through examples in oral and written communication	與病患進行口頭或書面溝通時, 能常規地將數字訊息, 例如風險值, 轉換為以舉例的方式說明
20	Routinely ensures that patients understand at minimum: 1) what their main problem is, 2) what is recommended that they do about it, and 3) why this is important	常規地確認病患至少了解到 1) 他們最主要的問題是甚麼, 2) 他們被建議要做甚麼, 3) 為什麼這個建議是重要的
21	Routinely writes in English at approximately the 5th-6th grade reading level	常規地使用約五、六年級能閱讀的程度來書寫資訊給病患
22	Consistently writes or re-writes (“translates”) unambiguous medication instruction when called for during regular duties	當有需要時, 寫或重寫(翻譯)不明確的用藥指示
23	Routinely conducts medication reconciliation with patients, including use of “brown bag” medication reviews, when called for during regular duties	在職務上需要時, 會常規地與病患核對其用藥情形, 包含讓病患將所使用的藥物和營養補充品帶來檢查與討論
24	Consistently uses a “universal precautions” approach to oral and written communication to patients	持續地採用通用守則來跟病患進行口頭或書面溝通
25	Consistently elicits questions from patients through a “patient-centered” approach (e.g., “what questions do you have?”, rather than “do you have any questions?”)	持續地透過以病患為中心的方式, 引導病患發問, 例如用「你有甚麼問題?」而不是「你有任何問題嗎? 」
26	Consistently locates and uses literacy-appropriate patient education materials, when needed and available, to reinforce oral communication, and reviews such materials with patients underlining or highlighting key information	持續地尋找及應用適合病患閱讀的衛教文本來加強口語溝通, 並且能與病患一同討論或標示出重點
27	Routinely arranges for timely follow-up when communication errors are anticipated	當預期會發生溝通錯誤時, 能常規地安排合時的後續追蹤
28	Routinely documents in the medical record that a “teach back,” or closed communication loop technique has been used to check the patient’s level of understanding at the end of the encounters	常規地在病歷上紀錄, 與病患會面互動結束前, 應用了回覆示教或封閉迴路溝通技巧來確認病患的理解程度
29	Routinely encourages and facilitates patients to carry an updated list of their medication with them	常規地鼓勵並建議病患帶一份近期用藥清單
30	Routinely refers patients to appropriate community resources for enhancing literacy and/or health literacy skills [e.g., Adult Basic Literacy Education] within the context of the therapeutic relationship	在醫病關係前提下, 轉介病患到適合的社區資源來, 提升讀寫能力與/或健康識能(舉例: 成人基礎識讀教育)
31	Consistently treats the diagnosis of limited health literacy as “protected health information” requiring specific “release of information” for disclosure	持續地將被認定為健康識能不足是受保護的資料, 需要特殊要求釋出訊息時才能揭露
32	Routinely anticipates and addresses navigational barriers within health care systems and shares responsibility with patients for understanding and navigating systems and processes; attempts to make systems and processes as transparent as possible	常規地預期及強調搜尋健康照護系統的阻礙, 分擔病患瞭解及搜尋系統和過程的責任, 盡可能讓系統和過程透明化

Finally, the panelists suggested a Likert scale format to measure health literacy competencies, either self-administered or observational.

## Discussion

This study employed a modified Delphi process to adapt a set of evidence-based health literacy knowledge, attitudes, and skills to the contexts of Taiwan’s healthcare delivery system and to guide the development of health literacy curricula for health professionals in Taiwan. Moreover, we utilized the Q-sorting technique to assign different degrees of importance to health literacy practices so as to inform healthcare providers and organizations of practices that are most beneficial to patients in alleviating barriers due to inadequate health literacy.

In this study, 91.9% consensus was achieved regarding the appropriateness and importance of 57 out of 62 health literacy competency items among a panel of Taiwanese experts. A previous European study had an expert group census of 90% [14]. Altogether, the results suggest a high level of expert agreement geographically across the US, Europe, and Taiwan. The understanding of health literacy competencies in terms of knowledge, attitude, and skills for health professionals shows similarities across Western and Eastern countries, despite the differences in healthcare delivery systems and sociocultural contexts. We further identified a core set of health literacy knowledge, attitudes, and skills required by health professionals. In total, 42 items reached a consensus, which collectively serve as a framework for guiding the development of curricula for enhancing health professionals’ health literacy competencies.

Health literacy practices are patient-centered strategies and behaviors that minimize the negative consequences of limited health literacy. The top-rated health literacy practice item in this study is “avoiding using medical jargon,” which is consistent with a previous American study wherein “avoiding medical jargon” was the second top-rated item. Health professionals commonly overestimate patients’ health literacy and unintentionally use medical jargon [25]. Use of medical jargon creates barriers to effective communication with patients, particularly among those with low health literacy, and limits patients’ ability to fully understand the medical information provided by health professionals [26]. Experts consistently agree that avoiding medical jargon is crucial and should be routinely practiced by health professionals in clinical encounters.

In Taiwan, health professionals learn medical knowledge by reading English or translated texts and most of the terminology has no common, plain language substitutes [27]. The process of learning, while increasing the medical expertise of health professionals, may lower their sensitivity to the use of medical jargon that is unfamiliar to most patients. Clearly, there is a need for health professionals to learn how to appropriately and clearly explain medical conditions. Simulations of clinical encounters with standardized patients, for example, may be helpful. Such simulations can increase medical professionals’ awareness of their use of medical jargon and offer them opportunities to practice communication in plain language. As gamification becomes a useful pedagogical tool in medical education [28], educators may develop digital-based learning games that allow students to be aware of medical jargon and learn the corresponding plain language or interpretation that they might replace.

“Teach-back” was rated the number one health literacy practice in an American study [16]. In contrast, our panelists rated the practice as the seventh. Research suggests that teach-back is an effective and feasible method to enhance patients’ health literacy and improve patients’ health outcomes [29, 30]. Patients reported satisfaction with their interactions with health professionals when the teach-back methods are applied. Health professionals also perceived improvement in their health communication with patients. However, there is concern that teach-back may evoke patients’ adverse emotions, even stigma [29]. It is likely that this concern may be more prominent, and the teach-back may be less appropriate and effective, in a collectivist culture. This may explain the lower rating of the teach-back practice by a Taiwanese Delphi panel in comparison to the result of a U.S study.

Given the strong evidence supporting the advantages of teach-back, the challenges of health professionals’ training include educating health professionals not only on routinely practicing teach-back techniques but also on methods to avoid making patients suspect their learning ability or feel stressed as if they were taking a test. The Agency for Healthcare Research and Quality [31] unveiled a set of toolkits, such as asking for non-shaming, open-ended questions or using plain languages, that utilized teach-back more efficiently, and provided good training material resources.

It is also interesting to note that the eight top-rated health literacy practice items our panelists prioritized are not identical to the eight top-rated items identified in the previous American study. Notably, four out of eight items our panelists affirmatively rated in Group 1 were not found in the previous study, including “speaks slowly and clearly,” “using analogies and examples,” “avoiding idioms and metaphors,” “active listening,” and “ask patient’s preferred learning style in a non-shaming manner.” The differences may be due to variations in culture and health service delivery systems. Items related to avoiding idioms and metaphors in a non-shaming manner could be a cultural issue. Eastern cultures, which tend to be collective, are characterized by an indirect communication style and maintain interpersonal harmony [32]. This style is a less overtly conflictual or aggressive form of communication, and individuals are more likely to use idioms and metaphors that may hinder clear communication in clinical encounters. In terms of “speak slowly” and “active listening” items, it may reflect the global budget payment system of the National Health Insurance program in Taiwan that compels healthcare providers to have a high volume of clinical work, thus lacking the time and patience for clear communication [33]. Observing patients’ nonverbal behavior and decoding proverbs and metaphors appear even more critical in indirect communication culture. Accordingly, our panelists are especially aware of and address these practices for routine use across health professionals to increase the clarity of communication and help mitigate the negative effects of low health literacy.

Currently, shared decision-making has become a focus for promoting patients’ engagement and empowerment. It is not surprising to note that two items, “eliciting the full list of patient concerns” and “negotiating a mutual agenda,” are two of the eight top-rated items in the American study and our research. However, previous studies showed that patients might have the desire to participate in health discussions and decision-making, despite the stress they may feel due to insufficient medical knowledge. Health professionals are patients’ advocates and they should encourage and support patients to gain a full understanding of health information, options, concerns, and rights during the medical decision process. It is clear that success in adhering to health promotion and self-care management behaviors relies on clear communication and taking patients’ concerns and preferences into account. Accordingly, “eliciting the full list of patient concerns” and “negotiating a mutual agenda” are essential health literacy practices for clinical and public health professionals that should not be neglected.

This study replicated US consensus studies to refine and validate health literacy competencies for health professionals in Taiwan. More than 90% high agreement in American, European [14], and Asian research indicated that the items of Coleman et al. [13] can be applied as indicators of health literacy competencies and practices across diverse healthcare and cultural contexts. In contrast to previous studies that employed local expert opinions to develop health professionals’ health literacy competencies [18, 19], the advantage of this set of measurable health literacy knowledge, attitudes, skills, and practices for health professionals is that it is comprehensive and evidence-based and that it has achieved consensus across global expert panelists. Health profession educators should feel confident in using this set of competencies and practices as core learning objectives for developing health literacy training programs and selecting the contents to match the learning activities and expected outcomes [13]. Additionally, the translated practice items would be beneficial for health literacy training programs among Mandarin-speaking health professionals.

This study has several limitations. First, although the health literacy competencies and practices examined in this study have considerable validity as they are based on accumulated international evidence [13], the results of consensus can only represent expert opinion. Our findings do not reflect the opinions of healthcare professional students or patients. Second, although the experts on our panel had diverse disciplinary backgrounds, not all health professions and medical disciplines were represented in our study. It is possible that the consensus results may vary depending upon the disciplines and areas of expertise represented on the expert panel. Insufficient representation is a limitation of recruitment and increases the likelihood of selection bias. Third, the list of health literacy competency items in this study was based on a US study and a review of literature that may be dated. Future studies should consider bring the literature review up to date and incorporate new health literacy competencies and practices identified in more recent research.

## Conclusions

This consensus study used a modified Delphi method to appraise a set of health literacy competencies and practices derived from a US study for its appropriateness, importance, and prioritization for health professionals in Taiwan. The high consensus across global health literacy experts suggests that the selected core health literacy competencies and practices could be used as guidelines and clinical assessments across healthcare professions. This set of health literacy competencies can provide a sound basis for developing health professional curricula or continuing education programs to enhance health literacy competencies and clear communication practices.

### Supplementary Information

**Supplementary Material 1. **

## Data Availability

The datasets generated and/or analyzed during the current study are not publicly available due ethical consideration but are available from the corresponding author on reasonable request.
