# Supplementary material for: Health literacy competency requirements for health professionals: a Delphi consensus study in Taiwan
Source: BMC Med Educ. 2024 Mar 1;24:209. doi: 10.1186/s12909-024-05198-4 (PMC10907998; doi:10.1186/s12909-024-05198-4)
Supplement: Supplementary file 1 — Supplementary Material 1. [file 12909_2024_5198_MOESM1_ESM.docx]

Appendix Table 1. The appropriateness and importance of health literacy competencies for health professionals

| Health literacy competencies  Knowledge  Health professionals… | Appropriateness  Mean ± SD | Importance  Mean ± SD |
| --- | --- | --- |
| 1. knows one or more definitions of health literacy | 2.00±0.94 | 1.70±0.78 |
| 1. knows the basic literacy skill domains (reading, writing, speaking, listening, numeracy), and gives examples of health care related demands put on patients for each domain, including difficulties navigating health care systems. | 2.25±1.04 | 1.55±0.66 |
| 1. knows the difference between the ability to read, and reading comprehension, and why general reading levels do no not ensure patient understanding. | 1.85±0.96 | 1.48±0.80 |
| 1. knows that years of educational attainment is an inadequate marker for health literacy skills. | 1.70±0.90 | 1.45±0.74 |
| 1. knows which kinds of words, phrases, or concepts may be jargon to patients. | 1.20±0.40 | 1.10±0.30 |
| 1. estimates the prevalence of low literacy (or low health literacy) among Taiwanese adults, and knows that certain subgroups are at increased risk. | 2.10±0.88 | 1.60±0.58 |
| 1. knows that the average Taiwanese adult reads at an 8th–9th-grade reading level, but that most patient education materials are written at a much higher reading level. | 1.95±0.97 | 1.50±0.59 |
| 1. knows that cultural and linguistic differences between patients and health care professionals can magnify health literacy issues. | 1.40±0.73 | 1.15±0.35 |
| 1. knows that adults with low literacy tend to experience shame, and hide their lack of skills from health care professionals | 1.75±0.94 | 1.20±0.51 |
| 1. knows that “you can’t tell who has low health literacy by looking”. | 1.75±0.88 | 1.55±0.74 |

Appendix Table 1. (continues)

| Health literacy competencies  Knowledge  Health professionals… | Appropriateness  Mean ± SD | Importance  Mean ± SD |
| --- | --- | --- |
| 1. recognizes “red flag” behaviors which may suggest a patient has low health literacy. | 1.75±0.88 | 1.25±0.53 |
| 1. knows that tools are available for estimating individuals’ health literacy skills, but that routine screening for low health literacy has not been proven safe or acceptable. | 2.70±1.10 | 2.25±0.94 |
| 1. knows that health literacy is context-specific; individuals with high general literacy may have low health literacy. | 1.45±0.58 | 1.30±0.45 |
| 1. knows that health literacy may decrease during times of physical or emotional stress. | 1.75±0.82 | 1.50±0.74 |
| 1. knows that everyone, regardless of literacy level, benefits from and prefers clear *plain language* communication. | 1.85±0.79 | 1.80±0.81 |
| 1. knows that transition points, or “hand-offs” in health care (e.g., moving from in-patient to out-patient settings) are especially vulnerable to patient communication errors. | 1.80±0.87 | 1.30±0.55 |
| 1. knows rationale for, and principles underpinning the need for a *universal precautions* approach to all health communication interactions. | 1.75±0.76 | 1.45±0.49 |
| 1. knows best practice principles of *plain language* and *clear health communication* for oral and written communication. | 1.15±0.47 | 1.10±0.43 |
| 1. knows that patients learn best when a limited number of new concepts are presented at any given time. | 1.80±0.87 | 1.35±0.57 |

Appendix Table 1. (continues)

| Health literacy competencies  Knowledge  Health professionals… | Appropriateness  Mean ± SD | Importance  Mean ± SD |
| --- | --- | --- |
| 1. knows examples of the direct relationship between health literacy and    - knowledge about one’s chronic disease(s) and medications.    - adherence to medications and treatment plans.    - receipt of preventive health services.    - health outcomes or risk of harm. | 1.75±0.69 | 1.45±0.58 |
| 1. recognizes potential legal implications for inadequately conveying health information to patients with low literacy or health literacy. | 2.10±1.04 | 1.70±0.84 |
| 1. knows that low health literacy has been associated with excess healthcare costs. | 1.55±0.74 | 1.60±0.86 |
| 1. knows the rationale for and mechanics of using a teach back or “show me” technique to assess patient understanding. | 1.45±0.74 | 1.05±0.21 |
| 1. knows that community resources exist for helping adults improve their general literacy skills. | 2.05±0.86 | 1.80±0.74 |
| Health literacy competencies  Attitude  Health professionals… | Appropriateness  Mean ± SD | Importance  Mean ± SD |
| 1. expresses the attitude that effective communication is essential to the delivery of safe high-quality health care. | 1.70±0.90 | 1.40±0.58 |
| 1. exhibits the attitude that all patients are at risk for communication errors, and that one cannot tell who is at risk of communication errors simply by looking, or through typical health care interactions—a universal precautions approach is required with all patients. | 1.90±0.88 | 1.45±0.49 |

Appendix Table 1. (continues)

| Health literacy competencies  Attitude  Health professionals… | Appropriateness  Mean ± SD | Importance  Mean ± SD |
| --- | --- | --- |
| 1. expresses the attitude that because the “culture” of healthcare includes special knowledge, language, logic, experiences and explanatory models of health and illness, every patient encounter can be considered a cross-cultural experience. | 1.50±0.59 | 1.25±0.43 |
| 1. expresses acceptance of an ethical responsibility to facilitate the two-way exchange of information in “shared decision making” to the degree and at the level desired by the patient and their family. | 1.35-0.47 | 1.15±0.35 |
| 1. acknowledges patients’ autonomous right to both informed consent, and “informed refusal” of recommended evaluations or treatments. | 1.50±0.74 | 1.40±0.58 |
| 1. expresses empathy with patients’ potential sense of shame around low literacy (or health literacy) issues. | 1.75±0.94 | 1.40±0.58 |
| 1. expresses a non-judgmental non-shaming respectful attitude toward individuals with limited literacy (or health literacy) skills. | 1.45±0.80 | 1.25±0.53 |
| 1. expresses empathy with the common experience of the health care system as a confusing, stressful, frustrating, intimidating, and frightening physical and virtual environment for many patients. | 1.65±0.85 | 1.30±0.45 |
| 1. expresses the attitude that every patient has the right to understand their health care, and that it is the health care professional’s duty to elicit and ensure patients’ best possible understanding of their health care | 1.50±0.59 | 1.25±0.53 |
| 1. expresses the attitude that it is a responsibility of the health care sector to address the mismatch between patients’ and health care providers communication skills and tactics. | 2.70±0.843 | 2.25±0.88 |
| 1. expresses the attitude that it is a responsibility of all members of the healthcare team to be trained and proactive in addressing the communication needs of patients. | 1.45±0.66 | 1.40±0.66 |

Appendix Table 1. (continues)

| Health literacy competencies  Skill  Health professionals… | Appropriateness  Mean ± SD | Importance  Mean ± SD |
| --- | --- | --- |
| 1. demonstrates ability to use common familiar lay terms, phrases and concepts, and appropriately define unavoidable jargon, and avoid using acronyms in oral and written communication with patients. | 1.65±1.06 | 1.25±0.69 |
| 1. demonstrates ability to recognize, avoid and/or constructively correct the use of medical *jargon*, as used by others in oral and written communication with patients. | 1.65±1.06 | 1.25±0.69 |
| 1. demonstrates ability to follow best-practice principles of easy-to-read formatting and writing in written communication with patients | 1.55±0.86 | 1.30±0.55 |
| 1. demonstrates ability to recognize plain language principles in written materials produced by others | 1.30±0.71 | 1.25±0.69 |
| 1. demonstrates the ability to put information into context by using subject headings in both written and oral communication with patients. | 1.40±0.73 | 1.25±0.69 |
| 1. demonstrates ability to write in Chinese at approximately the 5th-6th grade reading level | 1.60±0.86 | 1.40±0.80 |
| 1. demonstrates the ability to perform Chinese-to-Chinese translation of information from a non-plain language format into a scientifically accurate low-literacy plain language format. | 1.30±0.71 | 1.15±0.35 |
| 1. demonstrates ability to speak slowly and clearly with patients. | 1.35±0.57 | 1.10±0.30 |
| 1. demonstrates ability to use verbal and non-verbal active listening techniques when speaking with patients | 1.25±0.53 | 1.10±0.43 |
| 1. demonstrates the ability to use action oriented statements to help patients know what they need to do. | 1.05±0.21 | 1.00±0.00 |
| 1. demonstrates ability to select culturally and socially appropriate and relevant visual aids, including objects and models, to enhance and reinforce oral and written communication with patients | 1.20±0.40 | 1.10±0.30 |

Appendix Table 1. (continues)

| Health literacy competencies  Skill  Health professionals… | Appropriateness  Mean ± SD | Importance  Mean ± SD |
| --- | --- | --- |
| 1. demonstrates ability to make instructions interactive, such that patients engage the information, to facilitate retention and recall. | 1.25±0.53 | 1.10±0.30 |
| 1. demonstrates ability to elicit the patient’s full set of concerns at the outset of the encounter. | 1.65±0.72 | 1.35±0.57 |
| 1. demonstrates ability to negotiate a mutual agenda for the encounter at the outset of the encounter. | 1.65±0.79 | 1.45±0.74 |
| 1. demonstrates ability to elicit patients’ prior understanding of their health issues in a non-shaming manner (e.g., asks “what do you already know about high blood pressure? ”). | 1.35±0.47 | 1.15±0.35 |
| 1. demonstrates ability to non-judgmentally elicit root causes of non-adherent health behaviors. | 1.15±0.35 | 1.00±0.00 |
| 1. demonstrates effective use of a teach back or “show me” technique for assessing patients’ understanding. | 1.30±0.71 | 1.10±0.30 |
| 1. demonstrates ability to “Chunk and check” by giving patients small amounts of information and checking for understanding before moving to new information | 1.50±0.67 | 1.15±0.35 |
| 1. demonstrates ability to effectively elicit questions from patients through a “patient-centered” approach (e.g., asks “what questions do you have?” rather than “do you have any questions?”). | 1.65±0.91 | 1.40±0.58 |
| 1. demonstrates ability to orally communicate accurately and effectively in patients’ preferred language, using medical interpreter services | 1.30±0.45 | 1.25±0.53 |
| 1. demonstrates ability to use written communication to reinforce important oral information. | 1.40±0.66 | 1.25±0.53 |
| 1. demonstrates ability to emphasize one to three “need-to-know” or “need-to- do” concepts during a given patient encounter. | 1.50±0.67 | 1.35±0.57 |

Appendix Table 1. (continues)

| Health literacy competencies  Skill  Health professionals… | Appropriateness  Mean ± SD | Importance  Mean ± SD |
| --- | --- | --- |
| 1. demonstrates the ability to convey numeric information, such as risk, using low numeracy approaches, such as through examples, in oral and written communication. | 1.60±0.73 | 1.40±0.49 |
| 1. demonstrates ability to write or re-write (“translate”) unambiguous medication instructions (e.g., “take 1 tablet by mouth every morning and evening for high blood pressure,” rather than “take one tablet by mouth twice daily.” | 1.30±0.45 | 1.20±0.40 |
| 1. demonstrates the ability to assess the usability of web-based patient resources. | 2.10±0.83 | 1.80±0.67 |
| 1. demonstrates ability to ask patients about their learning style preferences (e.g., ask patients, “what is the best way for you to learn new information?” | 1.60±0.66 | 1.35±0.47 |
| 1. demonstrates ability to use examples or analogies to improve patients’ comprehension. | 1.20±0.40 | 1.15±0.35 |
